# Increasing affective distance - leftward prism adaptation amplifies alexithymia in healthy females

**DOI:** 10.3389/fpsyg.2025.1666287

**Published:** 2025-10-14

**Authors:** Laura Culicetto, Massimo Mucciardi, Chiara Lucifora, Alessandra Falzone, Francesco Tomaiuolo, Angelo Quartarone, Carmelo Mario Vicario, Selene Schintu

**Affiliations:** ^1^IRCCS Centro Neurolesi “Bonino-Pulejo”, Messina, Italy; ^2^Department of Cognitive Psychological Pedagogical and Cultural Studies Sciences, University of Messina, Messina, Italy; ^3^Department of Philosophy and Communication, University of Bologna, Bologna, Italy; ^4^Department of Clinical and Experimental Medicine, University of Messina, Messina, Italy; ^5^Center for Mind/Brain Sciences (CIMeC), University of Trento, Rovereto/Trento, Italy; ^6^Department of Psychological and Brain Sciences, The George Washington University, Washington, DC, United States

**Keywords:** emotions, spatial attention, sex differences, affective processing, right hemisphere dysfunction

## Abstract

**Introduction:**

Emotional processing is linked with spatial attention, which prioritizes emotional stimuli over neutral ones. The interconnection between spatial and emotional processing may rely on the overlap between the networks underpinning such cognitive functions. Recent evidence has indeed identified a link between the rightward visuospatial bias exhibited by healthy individuals and the challenge in understanding emotional states, so-called alexithymia. However, while spatial attention has been manipulated by prism adaptation (PA), a well-known sensorimotor training, whether this is possible with emotional processing has never been investigated.

**Methods:**

Ninety-five participants completed alexithymia questionnaires, Toronto Alexithymia Scale (TAS-20) and Perth Alexithymia Questionnaire (PAQ), before and after a single session of either leftward or rightward deviating PA.

**Results:**

While both males and females showed the expected sensorimotor aftereffect solely leftward PA modulated alexithymia scores, and it did so only for women. The results indicate that leftward PA not only affects visuospatial performance, but also emotional processing, particularly in how individuals perceive and interpret emotional proximity and distance.

**Discussion:**

Alexithymia may be, therefore, metaphorically linked to impaired perception of emotional closeness and remoteness. These findings suggest that PA may modulate emotional capacities in a sex-dependent manner, offering insights into its therapeutic potential while also highlighting the need for caution as prolonged PA-based interventions may affect emotional well-being.

## 1 Introduction

The relationship between spatial attention and emotion processing has been a key area of research.

Emotional stimuli attract spatial attention (Compton, 2003; Yiend, 2010), however, while fearful cues enhance attentional spatial resolution, they also negatively affect the temporal one emphasizing the strong interaction between emotion and attention (Bocanegra and Zeelenberg, 2021). Interestingly, the right hemisphere is relatively dominant in both emotional and spatial attention processing. This includes its role in conscious and unconscious emotional processing, attentional biases toward emotional stimuli, and the interplay between emotional valence and spatial attention (Armaghani et al., 2014; Hartikainen, 2021; Schwartz et al., 1979).

The relative dominance of the right hemisphere in attention is believed to be responsible for the inherent leftward bias, generally exhibited by young healthy individuals, known as pseudoneglect (Brooks et al., 2014; Jewell and McCourt, 2000). Pseudoneglect is opposite in terms of direction to the well-known pathological rightward bias observed in neglect patients. Right hemisphere disfunction, following for example a stroke, can indeed cause neglect - the failure to direct attention to the contralesional, left side, of space (Vallar, 1993). Interestingly, neglect-like behavior can be modeled in intact brains by temporary and reversibly hypo-activating the right parietal cortex, a crucial node of the attentional network (Behrmann et al., 2004; Malhotra et al., 2009). This can be achieved with either inhibitory transcranial magnetic stimulation (TMS; Fierro et al., 2001; Salatino et al., 2014; Schintu et al., 2021) or prism adaption (PA; Crottaz-Herbette et al., 2014; Schintu et al., 2022; Schintu et al., 2023). PA is a sensorimotor training that, by shifting vision laterally, recalibrates the sensorimotor coordinates and affects cognition in both neglect patients and healthy individuals (Bultitude et al., 2013; Michel, 2016; Redding and Wallace, 2006; Stratton, 1896).

Right hemisphere dysfunction has been also linked to alteration of emotional processing, known as alexithymia (Aftanas and Varlamov, 2007). Alexithymia is characterized by difficulty in identifying and describing one’s own feelings and emotions (Taylor and Bagby, 2012). Whether alexithymia represents a stable personality trait or a transient state remains a topic of ongoing debate in the literature. Longitudinal and clinical studies support both perspectives. On the one hand, trait-level alexithymia has been described as relatively stable and potentially rooted in neurobiological or developmental factors (e.g., Porcelli et al., 1996; Salminen et al., 1994; Schmidt et al., 1993; Taylor et al., 1997). On the other hand, several studies have shown that alexithymia levels can fluctuate and may be influenced by affective state or psychopathology, such as depression, anxiety, or substance use disorders (Haviland et al., 1994; Honkalampi et al., 2001). This distinction between trait and state (Martínez-Sánchez et al., 2003) suggests that while alexithymia may exhibit moderate to high relative stability over time, it is not entirely immune to short-term modulations, particularly in specific contexts or populations. State-dependent changes have been observed in response to acute stressors or psychopathological episodes (Cruise and Becerra, 2018; Messina et al., 2014).

Namely, right hemisphere dysfunctions have been associated with heightened levels of alexithymia (Lumley and Sielky, 2000; Spalletta et al., 2001). Interestingly, disruption in right hemisphere emotional processing may even lead to challenges in emotional speech emphasizing that language functions alone are insufficient without the right hemisphere’s contribution to emotional integration (Tabibnia and Zaidel, 2005; Sifneos, 1988; Zeitlin et al., 1989). The network underpinning alexithymia and the one responsible for spatial attention, particularly in terms of hemispheric lateralization, do overlap. Similar to the anatomically defined brain lesion network implicated in visuospatial awareness (Doricchi and Tomaiuolo, 2003; Doricchi et al., 2008), alexithymia has been associated with decreased activation in the right inferior parietal cortex and the right prefrontal cortex, alongside increased activation in the left inferior parietal cortex when compared to non-alexithymic individuals (Kano et al., 2003; Sturm and Levenson, 2011). This overlap suggests that dysfunctions in the right hemisphere, which impair emotional processing, may concurrently affect visuospatial biases. This commonality is further supported by the link between alexithymia and a rightward visuospatial shift observed by our laboratory in a large group of healthy individuals (Vicario et al., 2021). Namely, we found that the higher was the alexithymia score, as quantified by the Toronto Alexithymia Scale (TAS-20), the greater was the rightward bias those participants exhibited, as measured by the line bisection task. This was an interesting finding that brought up an even more intriguing question – can we manipulate alexithymia level as we manipulate visuospatial biases? Indeed, while visuospatial attention has been successfully modified in healthy individuals (Colent et al., 2000; McIntosh et al., 2023; Michel et al., 2003; Bultitude and Woods, 2010; Fortis et al., 2011; Schintu et al., 2014, 2018) whether the same can be done to the ability of verbalizing emotions (i.e., alexithymia level) has never been investigated.

As modulatory technique we chose PA that, according to the deviation of the visual displacement, reduces the pathological rightward bias in neglect patients (right PA; Crottaz-Herbette et al., 2017; Gammeri et al., 2024; Newport and Schenk, 2012; Rossetti et al., 1998) and induces a rightward bias in healthy individuals (left PA; Colent et al., 2000; Crottaz-Herbette et al., 2017; McIntosh et al., 2019; Schintu et al., 2014, 2017) by modulating the interhemispheric balance (Pisella et al., 2006). Another reason that guided our choice of PA as interventional tool is its wide-ranging aftereffects, which extend beyond spatial cognition. It has been indeed shown to effectively modulate performance in different cognitive domains, such as learning (Schintu et al., 2018) and memory (Turriziani et al., 2024) as well as language-related processes, namely phonemic fluency (Turriziani et al., 2021). Remarkably, it has been even found to influence obsessive behavior, thus extending its effect to the psychiatric domain (Magnani et al., 2022). The present study investigated whether PA-induced modulation could extend to emotional and affective processing, and thus whether it could be a possible tool to temporary influence emotional self-awareness mechanisms. Since healthy individuals generally exhibit a small but significant rightward bias in spatial judgments after adaptation to left PA (for example Colent et al., 2000; McIntosh et al., 2023; Michel et al., 2003; Schintu et al., 2014, 2017) we hypothesized left PA to increase alexithymia levels in healthy individuals. This prediction aligns with prior evidence indicating a positive relationship between rightward bias and alexithymia scores (Vicario et al., 2021). On the other hand, we anticipated that right PA, commonly used as a “control” for left PA in experimental paradigms (see Schintu et al., 2017), would have no effect or eventually reduce alexithymia levels. Finally, it is well known that men and women differ in their emotional communication skills (Buck et al., 1974) along with reported sex-based differences in hemispheric lateralization (Hall and Matsumoto, 2004; Kesler-West et al., 2001; Killgore and Yurgelun-Todd, 2001; Williams et al., 2005). While some research highlights a right-lateralized amygdala activation in males and a more bilateral or left-dominant pattern in females (Cahill et al., 2001; Schneider et al., 2011), meta-analyses have yielded inconsistent results, suggesting that emotional lateralization may vary by brain region and stimulus valence (Fusar-Poli et al., 2009; Wager et al., 2003). Electrophysiological studies also indicate greater right-hemisphere dominance in men and more bilateral or left-lateralized activation in women during emotional tasks (Arnone et al., 2011; Gasbarri et al., 2007; Proverbio et al., 2006). In light of these findings, we investigated the potential modulatory effect of PA separately in female and male participants.

In summary, we hypothesized that left-deviating PA would increase alexithymia levels in healthy individuals, whereas right-deviating PA would have no effect or, if anything, reduce alexithymia levels. Furthermore, given evidence of sex-related differences in emotional communication skills and hemispheric lateralization, we explored whether PA-induced changes might differ between males and females. Since PA acts on interhemispheric balance and males and females differ in the cerebral lateralization (Hall and Matsumoto, 2004; Kesler-West et al., 2001; Killgore and Yurgelun-Todd, 2001; Williams et al., 2005), we expected PA to differentially modulate emotional processing as a function of sex.

Understanding the interaction between visuospatial processing and emotional regulation mediated by hemispheric lateralization and sensorimotor recalibration through PA, would be useful to guide interventions for psychological distress.

## 2 Materials and methods

### 2.1 Participants

A total of 95 right-handed participants (50 females), between 18 and 35 years of age (mean age 23.2 ± standard deviation (SD) 3,97 years), with normal or corrected-to-normal vision participated in the study. All participants self-reported no history of neurological or psychiatric disorders and no use of medications affecting the central nervous system. Participants were randomly assigned to the two PA conditions (leftward and rightward) to avoid potential order bias. The groups were not matched a *priori* for baseline alexithymia scores (right-deviating PA: *N* = 49, 25 females, mean age: 23.42 ± SD 3.74 years; left-deviating PA: *N* = 46, 25 females, mean age: 22.95 ± SD 4.24 years). Participants were recruited through the University of Messina, primarily via university-wide announcements and departmental e-mailing lists. Participation was voluntary, and no financial compensation was provided. Written informed consent was obtained from all participants prior to participation and no financial compensation was provided. The study was approved by the local ethics committee of the Department of Cognitive, Psychological, Educational, and Cultural Studies, University of Messina, Italy (protocol n. COSPECS_4_2022) on 22 April 2022 and was conducted in accordance with the principles outlined in the Declaration of Helsinki.

### 2.2 Procedure

**FIGURE 1:**
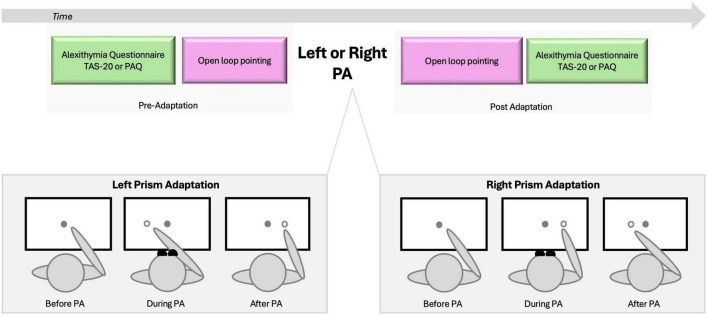
Experimental design. Upper panel illustrates the sequence of tasks. Alexithymia assessment (TAS-20 or PAQ) and open loop pointing were administered before and after adaptation to rightward or leftward prism adaptation. To eliminate any visual feedback, participants were unable to see their hands in the starting position or during the initial third of the pointing movement. Lower panel illustrates the three stages of left and right prism adaptation (PA). Before PA, the participants’ pointing performance aligns with the target. During PA, participants wear wedge prism glasses that deviate vision rightward (Left PA) or leftward (Right PA), and when asked to point to the target they show an error in the same direction of the PA deviation. Such error will be compensated as the pointing goes and participants will be able toward the end of training to point accurately to the target; they will be adapted to PA. Post PA, wedge glasses are removed and when participants are asked to point to the target they will show the sensorimotor after-effect, which is a pointing error in the direction opposite to the PA one (Schintu et al., 2020).

Following the informed consent procedure, participants completed the Edinburgh Handedness Inventory to quantify handedness (Oldfield, 1971). Before and after the single session of either right-deviating or left-deviating PA, they completed the Toronto Alexithymia Scale (TAS-20) and Perth Alexithymia Questionnaire (PAQ) (Figure 1). The order of administration of the two questionnaire was counterbalanced to prevent any potential learning effects due to close temporal proximity in which those were completed. Additionally, participants performed the open-loop pointing task (Figure 1) as part of the experimental protocol. For both the open-loop pointing and PA procedures, participants were comfortably seated in front of a horizontal board with their heads supported by a chinrest. Three circular targets (8 mm in diameter) were positioned on the board at 0° (center), −10° (left), and +10° (right) relative to the body midline, at approximately 57 cm from the participants’ nasion. Participants rested their left hand on the left thigh and pointed with their right index finger. The starting position for the right index finger was a 1.5 cm diameter Velcro^®^ pad located near the participant’s chest at the midline.

### 2.3 Prism adaptation

The PA procedure followed protocols similar to those used in previous studies (e.g., Schintu et al., 2014, 2018). Participants wore prism goggles (25 diopters) that shifted the visual field either leftward (left PA) or rightward (right PA). They performed 150 pointing movements to the right (+10°) and left (−10°) targets in a pseudorandom order, as verbally cued. Participants were instructed to extend their index finger in a single, continuous movement at a fast but comfortable speed. To ensure proper adaptation, participants were unable to see their hands in the starting position or during the initial third of the pointing movement (Figure 1).

### 2.4 Open-loop pointing

The open-loop pointing task assessed the sensorimotor shift induced by PA. Participants performed six pointing movements toward a central target (0°) without visual feedback and were instructed to maintain a consistent speed throughout the movement. Prior to each trial, participants were directed to visually fixate on the central target, subsequentially close their eyes, point toward the target while their eyes remained closed, and then return their finger to the starting position. To eliminate any visual feedback regarding the landing position of the finger, a cardboard baffle obscured the participant’s arm after they had looked at the target and closed their eyes. The baffle was removed only after the finger was returned to the starting pad, allowing the target to be visible for the subsequent movement (Schintu et al., 2018). The experimenter recorded the landing position of the participant’s finger with a measurement accuracy of 0.5 cm.

### 2.5 Alexithymia assessment

To assess both stable and context-sensitive components of alexithymia, we administered two complementary questionnaires, each grounded in distinct theoretical frameworks. The TAS-20 is based on the original conceptualization of alexithymia as a stable personality trait, characterized by difficulties in identifying and describing feelings and a tendency toward externally oriented thinking (Bagby et al., 1994; Taylor et al., 1997). In contrast, the PAQ is rooted in the Attention-Appraisal Model (Preece et al., 2017), which reconceptualizes alexithymia as a multidimensional construct involving deficits in emotional attention and appraisal. The PAQ was developed to address limitations of earlier measures by distinguishing between difficulties related to positive and negative affect, as well as between emotional awareness and expression (Preece et al., 2018). This dual-assessment strategy enabled us to capture both the trait-like and potentially state-sensitive aspects of alexithymia, thereby offering a broader and more nuanced perspective on potential modulation effects induced by PA.

#### 2.5.1 The 20-item Toronto Alexithymia Scale (TAS-20)

The TAS-20 is a widely recognized instrument for assessing alexithymia and includes three scales: difficulty identifying feelings (DIF; seven items, e.g., “I am often confused about what emotion I am feeling”), difficulty describing feelings (DDF; five items, e.g., “It is difficult for me to find the right words for my feelings”), and externally oriented thinking (EOT; eight items, e.g., “I prefer to just let things happen rather than to understand why they turned out that way”). The responses were measured using a five-point Likert scale, ranging from 1 (strongly disagree) to 5 (strongly agree). According to Bagby et al. (1994) and Taylor et al. (1997), the TAS-20 utilizes three cut-off scores for the classification of individuals: alexithymic subjects (≥61), borderline (score range between 51–60), and non-alexithymic subjects (≤50). In this study, the Italian version of the TAS-20 was used, which was validated by Bressi et al. (1996), Cronbach’s alpha: 0.75)

#### 2.5.2 Perth alexithymia questionnaire (PAQ)

The PAQ (Preece et al., 2018) is a 24-item self-report measure. Five subscales can be derived from the PAQ which capture the facets of alexithymia across positive and negative emotions: difficulty identifying negative feelings (N-DIF; e.g., “When I feel bad, I can’t tell if I’m sad, angry, or scared”), difficulty identifying positive feelings (P-DIF; e.g., “When I’m feeling good, I can’t tell if I’m happy, excited, or enjoying myself”), difficulty describing negative feelings (N-DDF; e.g., “When I feel bad, I can’t find the right words to describe those feelings”), difficulty describing positive feelings (P-DDF; e.g., “When I feel good, I may not find the right words to describe those feelings”) and oriented thinking outward (G-EOT; e.g., “I tend to ignore how I feel”). These five subscales can be summed to derive several composite scores, including the overall alexithymia total score. Subjects answer each item on a 7-point Likert scale, ranging from 1 (strongly disagree) to 7 (strongly agree), with higher scores indicating greater levels of alexithymia.

### 2.6 Statistical analysis

To ensure comparability of scores from the two alexithymia scales, data from each scale were normalized to their respective maximum score (i.e., TAS-20: 100; and PAQ: 168) and subsequently multiplied by 100. Statistical analyses were conducted using JASP (version 0.19.3) with a significance level set at α = 0.05. All results are presented as means accompanied by the within-subjects standard error of the mean (SEM). Effect sizes are reported for any significant effects identified.

For the Alexithymia scores, we used non-parametric tests since the data were not normally distributed as assessed by the Shapiro-Wilk test (*p* < 0.05). Baseline differences in alexithymia between males and females were tested with Mann–Whitney U tests. To assess the effects of PA on alexithymia, we compared post–pre change scores between left and right PA groups separately for each sex, also using Mann–Whitney U tests. Additional Mann–Whitney U tests were conducted to exclude potential order-of-administration effects of the two questionnaires.

To examine construct consistency, convergent validity between TAS-20 and PAQ subscales was tested with Spearman’s rank correlations. Correlations between alexithymia levels and sensorimotor aftereffects, as well as between alexithymia and handedness, were also assessed using Spearman’s (or Pearson’s, where normality was met) correlations.

For the open-loop pointing task, sensorimotor shifts were analyzed with independent-samples *t*-tests comparing left versus right PA groups separately for males and females. Finally, to verify that participants adapted to comparable levels, independent-samples *t*-tests were used to compare the absolute magnitude of the sensorimotor aftereffect between left and right PA, again reported separately for each sex.

## 3 Results

### 3.1 Alexithymia questionnaires

To quantify changes in alexithymia levels, mean scores for the PAQ and TAS-20 were calculated across pre- and post-assessment phases, with respect to the PA condition (left or right) and participant sex (Table 1). For each group, the alexithymia score before PA was subtracted from the post-PA score to obtain an alexithymia level. Negative values indicated a relative decrease from the baseline, and positive values indicated a relative increase.

**TABLE 1:** Meanscores for PAQ and TAS-20 by PA condition, order, and sex.

PA condition	Order	Sex	Pre (baseline)	Post (after PA)
Left PA	PAQ → TAS-20	Overall	67.66 (PAQ pre)	44.66 (TAS-20 post)
		Female	55.78 (PAQ pre)	40.0 (TAS-20 post)
		Male	84.3 (PAQ pre)	51.2 (TAS-20 post)
Right PA	PAQ → TAS-20	Overall	67.41 (PAQ pre)	43.87 (TAS-20 post)
		Female	75.33 (PAQ pre)	48.16 (TAS-20 post)
		Male	59.5 (PAQ pre)	39.58 (TAS-20 post)
Left PA	TAS-20 → PAQ	Overall	46.05 (TAS-20 pre)	74.5 (PAQ post)
		Female	50.45 (TAS-20 pre)	91.55 (PAQ post)
		Male	41.63 (TAS-20 pre)	57.45 (PAQ post)
Right PA	TAS-20 → PAQ	Overall	43.24 (TAS-20 pre)	66.44 (PAQ post)
		Female	42.84 (TAS-20 pre)	64.92 (PAQ post)
		Male	43.66 (TAS-20 pre)	68.08 (PAQ post)

We compared the alexithymia level between the left and right PA groups to investigate the effect of PA-induced alexithymia modulation. Given prior evidence that PA acts on interhemispheric balance and that males and females differ in hemispheric lateralization and emotional processing, we hypothesized that PA effects might differ based on sex. For this reason, we stratified the analyses by sex, independently of baseline difference. To confirm this assumption, we first tested whether baseline alexithymia levels differed between males and females. A Mann–Whitney U test revealed no significant sex difference at baseline (*U* = 1160.000, *p* = 0.797, *r* = 0.031), indicating that any observed modulation following PA cannot be attributed to pre-existing differences between groups.

**FIGURE 2:**
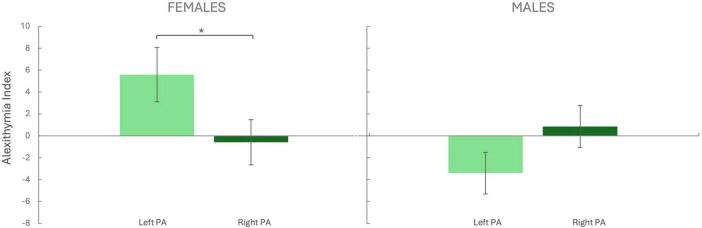
Alexithymia Index. The alexithymia index (Y-axis) was calculated for each group by subtracting the pre-PA score from the post-PA score. Negative values indicate a decrease, while positive values indicate an increase relative to baseline. Females Left PA (*N* = 25), Females Right PA (*N* = 25), Males Left PA (*N* = 21), Males Right PA (*N* = 24). PA, prism adaptation. Bars represent the Standard Error of the Mean (SEM). *p < 0.05.

For females, the Mann-Whitney U test revealed that the alexithymia level increased in the left PA group (5.58 ± 2.48) compared to the right PA group (−0.59 ± 2.07; *z* = *U* = 198.500, *p* = 0.028, *r* = −0.365, Figure 2). No difference was found for males between the left PA (−3.41 ± 1.89) and the right PA group (0.85 ± 1.91; *U* = 325.500, *p* = 0.097, *r* = 0.292).

As expected, solely left PA modulated cognition, and it did so by increasing the alexithymia level in females while no changes were produced in males.

To further control for a possible order-of-administration effect of the two alexithymia questionnaires, we conducted a Mann–Whitney U test, which revealed no significant difference (*U* = 101.000, *p* = 0.202, *r* = 0.312). This indicates that the modulation of the alexithymia level after left PA was not driven by one questionnaire being more sensitive than the other in detecting changes.

To address potential concerns regarding construct consistency, we conducted a convergent validity analysis between corresponding subscales of the TAS-20 and the PAQ. The convergent analysis (or convergent validity) is a method used in psychometrics and behavioral science to test whether two different measures that are theoretically supposed to measure the same construct are related in practice (Cheung et al., 2024). The results demonstrate strong and statistically significant correlations:

Right PA:

- TAS-DIF and PAQ-DIF (combined positive and negative): *r* = 0.7701, *p* < 0.0001
- TAS-DDF and PAQ-DDF (combined positive and negative): *r* = 0.7568, *p* < 0.0001
- TAS-EOT and PAQ-G-EOT: *r* = 0.7091, *p* < 0.0001

Left PA:

- TAS-DIF and PAQ-DIF (combined positive and negative): *r* = 0.6590; *p* < 0.001
- TAS-DDF and PAQ-DDF (combined positive and negative): *r* = 0.7029; *p* < 0.001
- TAS-EOT and PAQ-G-EOT: *r* = 0.5294; *p* < 0.001

These significant correlations provide evidence for the convergent validity of the two instruments and justify their combined use as a unified alexithymia level. While the PAQ offers a more nuanced structure, the strong alignment with the TAS-20 indicates that they measure a common underlying construct. Thus, the integration of these tools strengthens, rather than compromises, the validity of our pre/post comparison.

### 3.2 Open loop pointing task

The sensorimotor score before PA was subtracted from the post-PA score to obtain a sensorimotor index. Negative values indicated a relative leftward shift, and positive values indicated a relative rightward shift.

**FIGURE 3:**
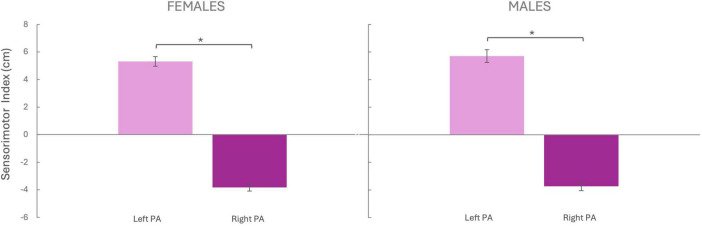
Sensorimotor Index. The sensorimotor index (Y-axis) was calculated by subtracting the pre-PA score from the post-PA score. Negative values indicate a leftward shift, while positive values indicate a rightward shift. Females Left PA (*N* = 25), Females Right PA (*N* = 25), Males Left PA (*N* = 21), Males Right PA (*N* = 24). PA, prism adaptation. Bars represent the Standard Error of the Mean (SEM). *p < 0.05

We compared the sensorimotor index between left and right PA groups to test the PA-induced sensorimotor shift. For females, the independent two-tailed *t*-test revealed that pointing performance differed between the left and right PA group [t(48) = –21.171 *p* < 0.001, *d* = 6.11; Figure 3], with the sensorimotor shift being rightward (5.32 ± 0.35 cm) after left PA and leftward (−3.83 ± 0.26 cm) after right PA. Similarly, for the male group, the pointing performance differed between the left and right PA group [t(43) = –17.461, *p* < 0.001, *d* = 5.33], with the sensorimotor shift being rightward (5.71 ± 0.46 cm) after left PA and leftward (−3.74 ± 0.31 cm) after right PA.

No sex differences were observed in the amount of sensorimotor adaptation, either after Left PA [t(44) = –0.684, *p* = 0.497, *d* = –0.023] or Right PA [t(47) = –0.224, *p* = 0.823, *d* = –0.064].

As expected, these results revealed that both males and females significantly adapted to both left and right PA, which induced the well-known leftward and rightward sensorimotor after-effects.

To check whether participants were adapted to comparable levels, we computed a *t*-test comparing the absolute value of the sensorimotor after effect between left and right PA, which resulted significant for both females [t(48) = –3.438, *p* = 0.001, *d* = –0.973] and males te [t(43) = –3.628, *p* < 0.001, *d* = –1.084] indicating that the sensorimotor aftereffect following left PA was larger than the one induced by right PA.

Finally, the Pearson correlation between the sensorimotor shift and the alexithymia level of the female group for whom left PA modulated it revealed no significant effect (*r* = −0.263, *p* = 0.204), meaning that the increase in alexithymia was not related to the amount of adaptation. For the same group, the Pearson correlation between the alexithymia level and handedness score revealed no significant correlation (*r* = 0.117, *p* = 0.576), meaning there was no significant relationship between the strength of handedness lateralization and alexithymia modulation.

## 4 Discussion

The present study investigated the impact of PA, a classical sensorimotor training that modulates spatial cognition, on alexithymia level. Building on previous research showing a tight correlation between alexithymia level and visuospatial bias (Vicario et al., 2021), as well as the effects of left PA on individuals with intact brain functions (Bultitude and Woods, 2010; Reed and Dassonville, 2014; Schintu et al., 2014) we hypothesized that left PA would increase alexithymia level, while no modulation was expected after right PA. Additionally, given the well-known difference in emotional processing and lateralization pattern between sexes, we explored the effect of PA separately for men and women. The results indicate that left PA increased alexithymia level among females, while no change was observed following right PA or among male participants.

A possible account for the effect of PA on emotional processing lies in the functional hemispheric asymmetry observed in spatial cognition and emotional processing along with the critical involvement of the parietal cortex in both functions as well as PA mechanism of action (Beraha et al., 2012; Crottaz-Herbette et al., 2014; Luauté et al., 2009; Shomstein, 2012). An original and influential model posits that PA shifts visuospatial attention by modulating the interhemispheric balance; namely by hypo-activating the parietal cortex ipsilateral to the PA deviation and hyper-activating the contralateral one (Boukrina and Chen, 2021; Pisella et al., 2006; Striemer and Danckert, 2010). Recent fMRI evidence from our group, in agreement with previous imaging findings in neglect patients (Saj et al., 2013), have instead shown no differential effect of PA across hemispheres in intact brains, but rather a bilateral decrease in connectivity following left PA (Schintu et al., 2020, 2022). Grounding on these recent findings and given that left PA induces a rightward visuospatial bias in healthy (Michel et al., 2003; Schintu et al., 2014, 2017) and, as shown here, also increases alexithymia, we reconduct such effect to the right hemisphere dominant role in spatial and emotional processing. Individuals with alexithymia as compared to non-alexithymia ones have indeed shown hypoactivation of the right parietal cortex, that correlated with alexithymia levels (Kano et al., 2003). Since the network associated with alexithymia overlaps with the visuospatial processing one, it is therefore plausible for left PA to exacerbate alexithymia levels by modulating the right hemisphere, which is dominant in emotional and spatial processing.

The absence of an effect following right PA may be attributed to the fact that significant behavioral modulation, such as alterations of visuospatial cognition, are rarely induced by right PA in healthy participants, to the extent that right PA has been often regarded as the gold-standard control condition for left PA. Numerous studies have indeed found that while left PA consistently induces significant visuospatial modulation, right PA frequently does not yield any noteworthy behavioral changes (Bultitude et al., 2013; Colent et al., 2000; Kano et al., 2003; Loftus et al., 2009; Michel et al., 2003; Reed and Dassonville, 2014; Schintu et al., 2014). The opposite, however, appears to hold true for pathological brains: only right PA ameliorates neglect behavior, while left PA leaves symptoms unchanged (Luauté et al., 2012). While this is the first evidence that PA can modulate emotional processing, it is possible, based on spatial-domain findings, that right PA could reduce alexithymia in individuals with pathologically high levels of the state/trait. This hypothesis however remains to be tested. Importantly, the present findings are not interpreted as evidence of a lasting transformation in personality traits. Instead, they are considered within a short-term modulation framework, consistent with the transient nature of PA effects. Previous studies (e.g., Schintu et al., 2014) have shown that PA can induce measurable changes in visuospatial attention that persist for approximately 40 min. Given the similarity in experimental design and timing, it is plausible that the observed changes in alexithymia reflect a comparable, state-like modulation rather than a stable trait alteration. Similarly, Fernández-Ruiz et al. (2004) identified a dual-phase decay pattern, with a rapid initial reduction in aftereffect magnitude within the first minute, followed by a more stable phase lasting up to 20 min. While longer-lasting effects have been observed, such as those persisting up to 7 days following extended adaptation sessions (Hatada et al., 2006), these typically require prolonged or repeated exposure. Thus, the present results align with the temporal window commonly reported for short-term PA-induced.

The observed sex differences likely stem from inherent variations in how cognitive and emotional networks interact, with women possibly exhibiting heightened sensitivity to the downstream effects of hemispheric modulation induced by PA. This sensitivity could be related to increased activation in emotional processing regions, such as the limbic system, as shown in brain imaging studies (e.g., Gur et al., 2002; Schintu et al., 2014). Additionally, emotional coping strategies differ between the sexes; women are generally more inclined to reflect upon and express their emotions, whereas men often tend to suppress or minimize emotional expression (Nolen-Hoeksema, 2012). This difference may make women more attuned to emotional shifts or challenges induced by interventions such as PA, whereas men’s tendency to suppress emotions might reduce their sensitivity to these changes. These variations in emotional responsiveness could explain the observed sex differences in alexithymia levels, with women showing greater sensitivity to changes in emotional processing. Importantly, no baseline differences in alexithymia were found between males and females, and no order-of-administration effect was detected between the two questionnaires. Furthermore, to our knowledge, no previous studies have reported differences in the sensitivity of the TAS-20 or PAQ across sexes. These controls suggest that the observed female-specific modulation is unlikely to be driven by baseline variability or measurement sensitivity.

Further support for the observed differences to be linked to higher-level emotional processing rather than low-level sensorimotor changes is given by the finding that both males and females successfully adapted to both left and right PA, with the two correlations found to be non-significant. This is not surprising given that the sensorimotor and cognitive aftereffects induced by PA rely on two functionally distinct subnetworks (Panico et al., 2022; Schintu et al., 2022). This indicates that the PA-induced changes may interact with emotional network differently across sexes. Finally, emotional distance and sensitivity to emotional cues are more readily modulated in females (Gur et al., 2002; Kring and Gordon, 1998), perhaps explaining why they show changes in alexithymia scores post-PA (Ooi et al., 2001).

Interestingly, the comparison of the absolute value of the sensorimotor aftereffect between left and right PA revealed a larger aftereffect following left PA in both males and females. We however believe that this asymmetry, although not consistently reported in previous studies (e.g., Schintu et al., 2017), does not affect the interpretation of our primary findings since sensorimotor and cognitive aftereffects rely on distinct mechanisms and typically do not correlate (Panico et al., 2020; Schintu et al., 2022). Moreover, participants of both sexes successfully adapted to both PA directions, and the modulation of alexithymia emerged only in females despite comparable sensorimotor adaptation across sexes. This suggests that the observed effects reflect higher-order emotional processes rather than low-level sensorimotor recalibration.

The neural reuse theory (Anderson, 2016), which posits neural circuits originally developed for specific functions can be repurposed for new tasks, may be a valuable framework for interpreting our findings. PA, traditionally used to recalibrate spatial attention, may also influence emotional processing through the mechanism of neural plasticity. In this context, PA might “repurpose” neural mechanisms associated with spatial perception to influence emotional regulation, particularly in individuals with alexithymia. This perspective offers insight into how interventions designed to manipulate spatial attention may yield downstream effects on emotional capacities, such as those related to alexithymia.

Furthermore, our results show that the modulation induced by PA has implications not only for spatial tasks, such as perceiving the length of lines (Cai et al., 2020) which relate to physical distance, but also for tasks involving affective processing. This suggests an interplay between the perception of physical and affective distances, how showcased in recent literature (Chen and Li, 2018) indicating that perception of spatial environment can reflect and influence emotional states and vice versa. Such interplay indicates that spatial cognition is not merely a navigational tool but also a framework through which we interpret emotional world. The perception of physical space around us is intricately linked with our emotional states. For example, a location that evokes feeling of emotional closeness may be perceived as physically nearer, than it actually is (Bar-Anan et al., 2007; Liberman et al., 2002). Conversely, entities or experiences associated with emotional distance may be perceived as physically more remote. This relationship demonstrates how spatial perception can influence feelings of proximity or separation. Psychological distance, defined as the extent of emotional connection experienced in interpersonal interactions (Wu and Bai, 2020), plays a crucial role in this context. In this regard, alexithymia can be metaphorically likened to a distorted perception of emotional proximity and distance. Just as PA affects spatial attention and perception of physical length, it may similarly impact how individuals interpret emotional proximity and distance. This analogy between the physical perception of space and the perception of affective distance in the context of alexithymia offers an intriguing perspective on how attentional processes might influence both our physical and emotional perceptions.

To summarize, our findings show that left PA increases alexithymia level solely in female participants, whereas right PA did not yield significant effects in either male or female participants. These findings not only suggest that PA, traditionally employed for spatial attention rehabilitation, may have broader applications such as influencing emotional capacities, but also underscore the importance of considering sex differences when designing PA-based interventions. Furthermore, given that alexithymia is associated with negative outcomes such as personal distress, somatic disorders, and anhedonia (Dubey and Pandey, 2003; Koppelberg et al., 2023), these results warn clinicians to consider these associations when developing repeated PA-based rehabilitation sessions. The potential side effects of prolonged PA use, particularly concerning emotional well-being, necessitate thorough psychological evaluation to mitigate any adverse impact.

Although an increase in alexithymia scores was observed in healthy females following left PA, and the effects of right PA on pathological brains have yet to be tested, we nonetheless urge caution, particularly in neglect rehabilitation, where patients often undergo repeated PA sessions. While the cognitive benefits of right PA are well established, its potential emotional side effects remain unknown. Overall, our findings highlight the importance of psychological monitoring during PA-based rehabilitation to mitigate any unintended effects on emotional processing.

Despite the relevance of these findings, some limitations of the current study should be acknowledged. First, the use of a composite alexithymia level derived from two questionnaires based on different theoretical models, the TAS-20 and the PAQ, may have limited the specificity of the results. While the dual-instrument approach was intended to provide a broader assessment of alexithymia, it may have obscured valence-specific or construct-specific differences. Future studies should consider measures aligned with a single theoretical framework. Second, the sample consisted exclusively of right-handed university students aged between 18 and 35 years. This sample may limit the generalizability of our findings to other age groups or clinical populations. Future research should include more diverse participants in terms of age, handedness, and educational background to assess the broader applicability of PA-induced emotional modulation. Finally, the absence of an emotional control task restricts the construct specificity of our findings, as it remains unclear whether the observed modulation reflects a selective effect on alexithymia or a broader influence on emotional processing. Future studies should incorporate additional emotional control measures to better disentangle PA-specific effects on alexithymia from more general effects on affective functions.

In conclusion, the present study highlights the potential for PA to modulate emotional processing, specifically alexithymia, in a sex-dependent manner. Overall, this study paves the way for a deeper understanding of how interventions targeting spatial attention may contribute to emotional regulation, providing promising insights into the novel therapeutic applications of PA.

## Data Availability

The raw data supporting the conclusions of this article will be made available by the authors, without undue reservation.

## References

[B1] Aftanas L. I. Varlamov A. A. (2007). Effects of alexithymia on the activity of the anterior and posterior areas of the cortex of the right hemisphere in positive and negative emotional activation. *Neurosci. Behav. Physiol.* 37 67–73. 10.1007/s11055-007-0151-z 17180321

[B2] Anderson M. L. (2016). Neural reuse in the organization and development of the brain. *Dev. Med. Child Neurol.* 58 (Suppl. 4), 3–6. 10.1111/dmcn.13039 27027600

[B3] Armaghani S. J. Crucian G. P. Heilman K. M. (2014). The influence of emotional faces on the spatial allocation of attention. *Brain Cogn.* 91 108–112. 10.1016/j.bandc.2014.09.006 25306560

[B4] Arnone B. Pompili A. Tavares M. C. Gasbarri A. (2011). Sex-related memory recall and talkativeness for emotional stimuli. *Front. Behav. Neurosci.* 5:52. 10.3389/fnbeh.2011.00052 21909326 PMC3164105

[B5] Bagby R. M. Parker J. D. Taylor G. J. (1994). The twenty-item Toronto Alexithymia Scale–I. Item selection and cross-validation of the factor structure. *J. Psychosom. Res.* 38 23–32. 10.1016/0022-3999(94)90005-1 8126686

[B6] Bar-Anan Y. Liberman N. Trope Y. Algom D. (2007). Automatic processing of psychological distance: Evidence from a Stroop task. *J. Exp. Psychol. Gen.* 136 610–622. 10.1037/0096-3445.136.4.610 17999574 PMC3161424

[B7] Behrmann M. Geng J. J. Shomstein S. (2004). Parietal cortex and attention. *Curr. Opin. Neurobiol.* 14 212–217. 10.1016/j.conb.2004.03.012 15082327

[B8] Beraha E. Eggers J. Hindi Attar C. Gutwinski S. Schlagenhauf F. Stoy M. (2012). Hemispheric asymmetry for affective stimulus processing in healthy subjects–a fMRI study. *PLoS One* 7:e46931. 10.1371/journal.pone.0046931 23056533 PMC3466188

[B9] Bocanegra B. R. Zeelenberg R. (2021). Emotional cues enhance the attentional effects on spatial and temporal resolution. *Psychon. Bull. Rev.* 18 1071–1076. 10.3758/s13423-011-0156-z 21901512 PMC3219868

[B10] Boukrina O. Chen P. (2021). Neural mechanisms of prism adaptation in healthy adults and individuals with spatial neglect after unilateral stroke: A review of fMRI studies. *Brain Sci.* 11:1468. 10.3390/brainsci11111468 34827467 PMC8615640

[B11] Bressi C. Taylor G. Parker J. Bressi S. Brambilla V. Aguglia E. (1996). Cross validation of the factor structure of the 20-item Toronto Alexithymia Scale: An Italian multicenter study. *J. Psychosom. Res.* 41 551–559. 10.1016/s0022-3999(96)00228-0 9032718

[B12] Brooks J. L. Della Sala S. Darling S. (2014). Representational pseudoneglect: A review. *Neuropsychol. Rev.* 24 148–165. 10.1007/s11065-013-9245-2 24414221

[B13] Buck R. Miller R. E. Caul W. F. (1974). Sex, personality, and physiological variables in the communication of affect via facial expression. *J. Pers. Soc. Psychol.* 30 587–596. 10.1037/h0037041 4455775

[B14] Bultitude J. H. Woods J. M. (2010). Adaptation to leftward-shifting prisms reduces the global processing bias of healthy individuals. *Neuropsychologia* 48 1750–1756. 10.1016/j.neuropsychologia.2010.02.024 20219496

[B15] Bultitude J. H. Van der Stigchel S. Nijboer T. C. (2013). Prism adaptation alters spatial remapping in healthy individuals: Evidence from double-step saccades. *Cortex* 49 759–770. 10.1016/j.cortex.2012.01.008 22386659

[B16] Cahill L. Haier R. J. White N. S. Fallon J. Kilpatrick L. Lawrence C. (2001). Sex-related difference in amygdala activity during emotionally influenced memory storage. *Neurobiol. Learn. Mem.* 75 1–9. 10.1006/nlme.2000.3999 11124043

[B17] Cai Y. C. Su X. Yang Y. M. Pan Y. Zhu L. Luo L. J. (2020). How does attention alter length perception? a prism adaptation study. *Front. Psychol.* 11:2091. 10.3389/fpsyg.2020.02091 32973630 PMC7461973

[B18] Chen H. Li S. (2018). Measuring the psychological distance between an organization and its members-the construction and validation of a new scale. *Front. Psychol.* 8:2296. 10.3389/fpsyg.2017.02296 29375427 PMC5767263

[B19] Cheung G. W. Cooper-Thomas H. D. Lau R. S. Wang L. C. (2024). Reporting reliability, convergent and discriminant validity with structural equation modeling: A review and best-practice recommendations. *Asia Pacific J. Manag.* 41 745–783. 10.1007/s10490-023-09871-y

[B20] Colent C. Pisella L. Bernieri C. Rode G. Rossetti Y. (2000). Cognitive bias induced by visuo-motor adaptation to prisms: A simulation of unilateral neglect in normal individuals? *Neuroreport* 11 1899–1902. 10.1097/00001756-200006260-00019 10884040

[B21] Compton R. J. (2003). The interface between emotion and attention: A review of evidence from psychology and neuroscience. *Behav. Cogn. Neurosci. Rev.* 2 115–129. 10.1177/1534582303255278 13678519

[B22] Crottaz-Herbette S. Fornari E. Clarke S. (2014). Prismatic adaptation changes visuospatial representation in the inferior parietal lobule. *J. Neurosci.* 34 11803–11811. 10.1523/JNEUROSCI.3184-13.2014 25164675 PMC6608412

[B23] Crottaz-Herbette S. Fornari E. Tissieres I. Clarke S. (2017). A brief exposure to leftward prismatic adaptation enhances the representation of the ipsilateral, right visual field in the right inferior parietal lobule. *eNeuro* 4:ENEURO.0310-17.2017. 10.1523/ENEURO.0310-17.2017. 28955725 PMC5615250

[B24] Cruise K. E. Becerra R. (2018). Alexithymia and problematic alcohol use: A critical update. *Addict. Behav.* 77 232–246. 10.1016/j.addbeh.2017.09.025 29107201

[B25] Doricchi F. Tomaiuolo F. (2003). The anatomy of neglect without hemianopia: A key role for parietal-frontal disconnection? *Neuroreport* 14 2239–2243. 10.1097/00001756-200312020-00021 14625455

[B26] Doricchi F. Thiebaut de Schotten M. Tomaiuolo F. Bartolomeo P. (2008). White matter (dis)connections and gray matter (dys)functions in visual neglect: Gaining insights into the brain networks of spatial awareness. *Cortex* 44 983–995. 10.1016/j.cortex.2008.03.006 18603235

[B27] Dubey A. Pandey R. (2003). *Mental health problems in alexithymia: Role of positive and negative emotional experiences.* Available online at: https://www.researchgate.net/publication/242655598 [accessed July 29, 2024].

[B28] Fernández-Ruiz J. Dıaz R. Aguilar C. Hall-Haro C. (2004). Decay of prism aftereffects under passive and active conditions. *Cogn. Brain Res.* 20 92–97. 10.1016/j.cogbrainres.2004.01.007 15130593

[B29] Fierro B. Piazza A. Brighina F. La Bua V. Buffa D. Oliveri M. (2001). Modulation of intracortical inhibition induced by low- and high-frequency repetitive transcranial magnetic stimulation. *Exp. Brain Res.* 138 452–457. 10.1007/s002210100728 11465743

[B30] Fortis P. Goedert K. M. Barrett A. M. (2011). Prism adaptation differently affects motor-intentional and perceptual-attentional biases in healthy individuals. *Neuropsychologia* 49 2718–2727. 10.1016/j.neuropsychologia.2011.05.020 21663753 PMC3137707

[B31] Fusar-Poli P. Placentino A. Carletti F. Allen P. Landi P. Abbamonte M. (2009). Laterality effect on emotional faces processing: Ale meta-analysis of evidence. *Neurosci. Lett.* 452 262–267. 10.1016/j.neulet.2009.01.065 19348735

[B32] Gammeri R. Schintu S. Salatino A. Vigna F. Mazza A. Gindri P. (2024). Effects of prism adaptation and visual scanning training on perceptual and response bias in unilateral spatial neglect. *Neuropsychol. Rehabil.* 34 155–180. 10.1080/09602011.2022.2158876 36652376

[B33] Gasbarri A. Arnone B. Pompili A. Pacitti F. Pacitti C. Cahill L. (2007). Sex-related hemispheric lateralization of electrical potentials evoked by arousing negative stimuli. *Brain Res.* 1138 178–186. 10.1016/j.brainres.2006.12.073 17274960

[B34] Gur R. C. Gunning-Dixon F. Bilker W. B. Gur R. E. (2002). Sex differences in temporo-limbic and frontal brain volumes of healthy adults. *Cereb. Cortex* 12 998–1003. 10.1093/cercor/12.9.998 12183399

[B35] Hall J. A. Matsumoto D. (2004). Gender differences in judgments of multiple emotions from facial expressions. *Emotion* 4 201–206. 10.1037/1528-3542.4.2.201 15222856

[B36] Hartikainen K. M. (2021). Emotion-Attention interaction in the right hemisphere. *Brain Sci.* 11:1006. 10.3390/brainsci11081006 34439624 PMC8394055

[B37] Hatada Y. Rossetti Y. Miall R. C. (2006). Long-lasting aftereffect of a single prism adaptation: Shifts in vision and proprioception are independent. *Exp. Brain Res.* 173 415–424. 10.1007/s00221-006-0381-2 16552560

[B38] Haviland M. G. Hendryx M. S. Shaw D. G. Henry J. P. (1994). Alexithymia in women and men hospitalized for psychoactive substance dependence. *Compr. Psychiatry* 35 124–128. 10.1016/0010-440x(94)90056-n 8187475

[B39] Honkalampi K. Koivumaa-Honkanen H. Tanskanen A. Hintikka J. Lehtonen J. Viinamäki H. (2001). Why do alexithymic features appear to be stable? A 12-month follow-up study of a general population. *Psychother. Psychosom.* 70 247–253. 10.1159/000056262 11509894

[B40] Jewell G. McCourt M. E. (2000). Pseudoneglect: A review and meta-analysis of performance factors in line bisection tasks. *Neuropsychologia* 38 93–110. 10.1016/s0028-3932(99)00045-7 10617294

[B41] Kano M. Fukudo S. Gyoba J. Kamachi M. Tagawa M. Mochizuki H. (2003). Specific brain processing of facial expressions in people with alexithymia: An H2 15O-PET study. *Brain* 126(Pt 6), 1474–1484. 10.1093/brain/awg131 12764066

[B42] Kesler-West M. L. Andersen A. H. Smith C. D. Avison M. J. Davis C. E. Kryscio R. J. (2001). Neural substrates of facial emotion processing using fMRI. *Brain Res. Cogn. Brain Res.* 11 213–226. 10.1016/s0926-6410(00)00073-2 11275483

[B43] Killgore W. D. Yurgelun-Todd D. A. (2001). Sex differences in amygdala activation during the perception of facial affect. *Neuroreport* 12 2543–2547. 10.1097/00001756-200108080-00050 11496145

[B44] Koppelberg P. Kersting A. Suslow T. (2023). Alexithymia and interpersonal problems in healthy young individuals. *BMC Psychiatry* 23:688. 10.1186/s12888-023-05191-z 37735376 PMC10515237

[B45] Kring A. M. Gordon A. H. (1998). Sex differences in emotion: Expression, experience, and physiology. *J. Pers. Soc. Psychol.* 74 686–703. 10.1037//0022-3514.74.3.686 9523412

[B46] Liberman N. Sagristano M. D. Trope Y. (2002). The effect of temporal distance on level of mental construal. *J. Exp. Soc. Psychol.* 38 523–534. 10.1016/S0022-1031(02)00535-8

[B47] Loftus A. M. Vijayakumar N. Nicholls M. E. (2009). Prism adaptation overcomes pseudoneglect for the greyscales task. *Cortex* 45 537–543. 10.1016/j.cortex.2007.12.011 19231481

[B48] Luauté J. Jacquin-Courtois S. O’Shea J. Christophe L. Rode G. Boisson D. (2012). Left-Deviating prism adaptation in left neglect patient: Reflexions on a negative result. *Neural Plast* 2012:718604. 10.1155/2012/718604 23050168 PMC3463195

[B49] Luauté J. Schwartz S. Rossetti Y. Spiridon M. Rode G. Boisson D. (2009). Dynamic changes in brain activity during prism adaptation. *J. Neurosci.* 29 169–178. 10.1523/JNEUROSCI.3054-08.2009 19129395 PMC6664918

[B50] Lumley M. A. Sielky K. (2000). Alexithymia, gender, and hemispheric functioning. *Compr. Psychiatry* 41 352–359. 10.1053/comp.2000.9014 11011831

[B51] Magnani B. Frassinetti F. Franceschini C. Dimaggio G. Musetti A. (2022). Right-deviating prismatic adaptation reduces obsessions in a community sample. *Front. Psychol.* 13:1025379. 10.3389/fpsyg.2022.1025379 36619054 PMC9811126

[B52] Malhotra P. Coulthard E. J. Husain M. (2009). Role of right posterior parietal cortex in maintaining attention to spatial locations over time. *Brain* 132(Pt 3), 645–660. 10.1093/brain/awn350 19158107 PMC2664449

[B53] Martínez-Sánchez F. Ato-García M. Ortiz-Soria B. (2003). Alexithymia–state or trait? *Span J. Psychol.* 6 51–59. 10.1017/s1138741600005205 12765051

[B54] McIntosh R. D. Brown B. M. A. Young L. (2019). Meta-analysis of the visuospatial aftereffects of prism adaptation, with two novel experiments. *Cortex* 111 256–273. 10.1016/j.cortex.2018.11.013 30530268

[B55] McIntosh R. D. Ten Brink A. F. Mitchell A. G. Jones H. Peng N. Thye M. (2023). A registered re-examination of the effects of leftward prism adaptation on landmark judgements in healthy people. *Cortex* 158 139–157. 10.1016/j.cortex.2022.11.003 36529083

[B56] Messina A. Beadle J. N. Paradiso S. (2014). Towards a classification of alexithymia: Primary, secondary and organic. *J. Psychopathol.* 20 38–49.

[B57] Michel C. (2016). Beyond the sensorimotor plasticity: Cognitive expansion of prism adaptation in healthy individuals. *Front. Psychol.* 6:1979. 10.3389/fpsyg.2015.01979 26779088 PMC4700133

[B58] Michel C. Pisella L. Halligan P. W. Luauté J. Rode G. Boisson D. (2003). Simulating unilateral neglect in normals using prism adaptation: Implications for theory. *Neuropsychologia* 41 25–39. 10.1016/s0028-3932(02)00135-5 12427563

[B59] Newport R. Schenk T. (2012). Prisms and neglect: What have we learned? *Neuropsychologia* 50 1080–1091. 10.1016/j.neuropsychologia.2012.01.023 22306519

[B60] Nolen-Hoeksema S. (2012). Emotion regulation and psychopathology: The role of gender. *Annu. Rev. Clin. Psychol.* 8 161–187. 10.1146/annurev-clinpsy-032511-143109 22035243

[B61] Oldfield R. C. (1971). The assessment and analysis of handedness: The Edinburgh inventory. *Neuropsychologia* 9 97–113. 10.1016/0028-3932(71)90067-4 5146491

[B62] Ooi T. L. May K. A. Gunther P. J. He Z. J. (2001). Prism adaptation effects on absolute distance judgment. *J. Vis.* 1:5. 10.1167/1.3.5

[B63] Panico F. Rossetti Y. Trojano L. (2020). On the mechanisms underlying prism adaptation: A review of neuro-imaging and neuro-stimulation studies. *Cortex* 123 57–71. 10.1016/j.cortex.2019.10.003 31759324

[B64] Panico F. Schintu S. Trojano L. (2022). Editorial: Uncovering the neural correlates of prism adaptation: Evidence from the brain network approach. *Front. Psychol.* 13:1076307. 10.3389/fpsyg.2022.1076307 36457912 PMC9706180

[B65] Pisella L. Rode G. Farnè A. Tilikete C. Rossetti Y. (2006). Prism adaptation in the rehabilitation of patients with visuo-spatial cognitive disorders. *Curr. Opin. Neurol.* 19 534–542. 10.1097/WCO.0b013e328010924b 17102690

[B66] Porcelli P. Leoci C. Guerra V. Taylor G. J. Bagby R. M. (1996). A longitudinal study of alexithymia and psychological distress in inflammatory bowel disease. *J. Psychosom. Res.* 41 569–573. 10.1016/s0022-3999(96)00221-8 9032720

[B67] Preece D. Becerra R. Allan A. Robinson K. Dandy J. (2017). Establishing the theoretical components of alexithymia via factor analysis: Introduction and validation of the attention-appraisal model of alexithymia. *Pers. Individ. Dif.* 119 341–352. 10.1016/j.paid.2017.08.003

[B68] Preece D. Becerra R. Robinson K. Dandy J. Allan A. (2018). The psychometric assessment of alexithymia: Development and validation of the perth alexithymia questionnaire. *Pers. Individ. Dif.* 132 32–44. 10.1016/j.paid.2018.05.011

[B69] Proverbio A. M. Brignone V. Matarazzo S. Del Zotto M. Zani A. (2006). Gender differences in hemispheric asymmetry for face processing. *BMC Neurosci.* 7:44. 10.1186/1471-2202-7-44 16762056 PMC1523199

[B70] Redding G. M. Wallace B. (2006). Prism adaptation and unilateral neglect: Review and analysis. *Neuropsychologia* 44 1–20. 10.1016/j.neuropsychologia.2005.04.009 15907951

[B71] Reed S. A. Dassonville P. (2014). Adaptation to leftward-shifting prisms enhances local processing in healthy individuals. *Neuropsychologia* 56 418–427. 10.1016/j.neuropsychologia.2014.02.012 24560913 PMC4512509

[B72] Rossetti Y. Rode G. Pisella L. Farné A. Li L. Boisson D. (1998). Prism adaptation to a rightward optical deviation rehabilitates left hemispatial neglect. *Nature* 395 166–169. 10.1038/25988 9744273

[B73] Saj A. Cojan Y. Vocat R. Luauté J. Vuilleumier P. (2013). Prism adaptation enhances activity of intact fronto-parietal areas in both hemispheres in neglect patients. *Cortex* 49 107–119. 10.1016/j.cortex.2011.10.009 22154751

[B74] Salatino A. Poncini M. George M. S. Ricci R. (2014). Hunting for right and left parietal hot spots using single-pulse TMS: Modulation of visuospatial perception during line bisection judgment in the healthy brain. *Front. Psychol.* 5:1238. 10.3389/fpsyg.2014.01238 25400612 PMC4215615

[B75] Salminen J. K. Saarijärvi S. Aäirelä E. Tamminen T. (1994). Alexithymia–state or trait? One-year follow-up study of general hospital psychiatric consultation out-patients. *J. Psychosom. Res.* 38 681–685. 10.1016/0022-3999(94)90020-5 7877122

[B76] Schintu S. Cunningham C. A. Freedberg M. Taylor P. Gotts S. J. Shomstein S. (2021). Callosal anisotropy predicts attentional network changes after parietal inhibitory stimulation. *Neuroimage* 226:117559. 10.1016/j.neuroimage.2020.117559 33189929 PMC7885523

[B77] Schintu S. Freedberg M. Alam Z. M. Shomstein S. Wassermann E. M. (2018). Left-shifting prism adaptation boosts reward-based learning. *Cortex* 109 279–286. 10.1016/j.cortex.2018.09.021 30399479 PMC7327780

[B78] Schintu S. Freedberg M. Gotts S. J. Cunningham C. A. Alam Z. M. Shomstein S. (2020). Prism adaptation modulates connectivity of the intraparietal sulcus with multiple brain networks. *Cereb. Cortex* 30 4747–4758. 10.1093/cercor/bhaa032 32313949 PMC7526755

[B79] Schintu S. Gotts S. J. Freedberg M. Shomstein S. Wassermann E. M. (2022). Effective connectivity underlying neural and behavioral components of prism adaptation. *Front. Psychol.* 13:915260. 10.3389/fpsyg.2022.915260 36118425 PMC9479732

[B80] Schintu S. Kravitz D. J. Silson E. H. Cunningham C. A. Wassermann E. M. Shomstein S. (2023). Dynamic changes in spatial representation within the posterior parietal cortex in response to visuomotor adaptation. *Cereb. Cortex* 33 3651–3663. 10.1093/cercor/bhac298 35989306 PMC10068280

[B81] Schintu S. Patané I. Caldano M. Salemme R. Reilly K. T. Pisella L. (2017). The asymmetrical effect of leftward and rightward prisms on intact visuospatial cognition. *Cortex* 97 23–31. 10.1016/j.cortex.2017.09.015 29078083 PMC5716840

[B82] Schintu S. Pisella L. Jacobs S. Salemme R. Reilly K. T. Farnè A. (2014). Prism adaptation in the healthy brain: The shift in line bisection judgments is long lasting and fluctuates. *Neuropsychologia* 53 165–170. 10.1016/j.neuropsychologia.2013.11.013 24291512

[B83] Schmidt U. Jiwany A. Treasure J. (1993). A controlled study of alexithymia in eating disorders. *Compr. Psychiatry* 34 54–58. 10.1016/0010-440x(93)90036-4 8425393

[B84] Schneider S. Peters J. Bromberg U. Brassen S. Menz M. M. Miedl S. F. (2011). Boys do it the right way: Sex-dependent amygdala lateralization during face processing in adolescents. *Neuroimage* 56 1847–1853. 10.1016/j.neuroimage.2011.02.019 21316467

[B85] Schwartz G. E. Davidson R. J. Maer F. (1979). Right hemisphere lateralization for emotion in the human brain: Interactions with cognition. *Science* 190 286–288. 10.1126/science.1179210 1179210

[B86] Shomstein S. (2012). Cognitive functions of the posterior parietal cortex: Top-down and bottom-up attentional control. *Front. Integr. Neurosci.* 6:38. 10.3389/fnint.2012.00038 22783174 PMC3389368

[B87] Sifneos P. E. (1988). Alexithymia and its relationship to hemispheric specialization, affect, and creativity. *Psychiatr. Clin. North Am.* 11 287–292. 3067225

[B88] Spalletta G. Pasini A. Costa A. De Angelis D. Ramundo N. Paolucci S. (2001). Alexithymic features in stroke: Effects of laterality and gender. *Psychosom. Med.* 63 944–950. 10.1097/00006842-200111000-00013 11719633

[B89] Stratton G. (1896). Some preliminary experiments on vision without inversion of the retinal image. *Psychol. Rev.* 3 611–617. 10.1037/h0072918

[B90] Striemer C. L. Danckert J. A. (2010). Through a prism darkly: Re-evaluating prisms and neglect. *Trends Cogn. Sci.* 14 308–316. 10.1016/j.tics.2010.04.001 20444640

[B91] Sturm V. E. Levenson R. W. (2011). Alexithymia in neurodegenerative disease. *Neurocase* 17 242–250. 10.1080/13554794.2010.532503 21432723 PMC3278303

[B92] Tabibnia G. Zaidel E. (2005). Alexithymia, interhemispheric transfer, and right hemispheric specialization: A critical review. *Psychother. Psychosom.* 74 81–92. 10.1159/000083166 15741757

[B93] Taylor G. J. Bagby R. M. (2012). “The alexithymia personality dimension,” in *The Oxford handbook of personality disorders*, ed. Widiger T. A. (Oxford: Oxford University Press), 648–673.

[B94] Taylor G. J. Bagby R. M. Parker J. D. A. (1997). *Disorders of affect regulation: Alexithymia in medical and psychiatric illness.* New York, NY: Cambridge University Press.

[B95] Turriziani P. Campo F. F. Bonaventura R. E. Mangano G. R. Oliveri M. (2024). Modulation of memory by prism adaptation in healthy subjects. *Sci. Rep.* 14:25358. 10.1038/s41598-024-77027-z 39455697 PMC11511821

[B96] Turriziani P. Chiaramonte G. Mangano G. R. Bonaventura R. E. Smirni D. Oliveri M. (2021). Improvement of phonemic fluency following leftward prism adaptation. *Sci. Rep.* 11:7313. 10.1038/s41598-021-86625-0 33790347 PMC8012568

[B97] Vallar G. (1993). “The anatomical basis of spatial hemineglect in humans,” in *Unilateral neglect: Clinical and experimental studies*, eds Robertson I. H. Marshall J. C. (Mahwah, NJ: Lawrence Erlbaum Associates, Inc.).

[B98] Vicario C. M. Martino G. Marcuzzo A. Craparo G. (2021). No evidence of perceptual pseudoneglect in alexithymia. *Brain Sci.* 11:376. 10.3390/brainsci11030376 33804270 PMC8001858

[B99] Wager T. D. Phan K. L. Liberzon I. Taylor S. F. (2003). Valence, gender, and lateralization of functional brain anatomy in emotion: A meta-analysis of findings from neuroimaging. *Neuroimage* 19 513–531. 10.1016/s1053-8119(03)00078-8 12880784

[B100] Williams L. M. Barton M. J. Kemp A. H. Liddell B. J. Peduto A. Gordon E. (2005). Distinct amygdala-autonomic arousal profiles in response to fear signals in healthy males and females. *Neuroimage* 28 618–626. 10.1016/j.neuroimage.2005.06.035 16081303

[B101] Wu Q. Bai L. (2020). The optimal integrated performance of person and organization: The comprehensive utility of PO bidirectional fit. *J. Beijing Instit. Technol.* 4 105–110. 10.15918/j.jbitss1009-3370.2015.0415

[B102] Yiend J. (2010). The effects of emotion on attention: A review of attentional processing of emotional information. *Cogn. Emot.* 24 3–47. 10.1080/02699930903205698

[B103] Zeitlin S. B. Lane R. D. O’Leary D. S. Schrift M. J. (1989). Interhemispheric transfer deficit and alexithymia. *Am. J. Psychiatry* 146 1434–1439. 10.1176/ajp.146.11.1434 2817114

